# Sub-phonon-period compression of electron pulses for atomic diffraction

**DOI:** 10.1038/ncomms9723

**Published:** 2015-10-27

**Authors:** A. Gliserin, M. Walbran, F. Krausz, P. Baum

**Affiliations:** 1 Ludwig-Maximilians-Universität München, Am Coulombwall 1, 85748 Garching, Germany; 2 Max-Planck-Institute of Quantum Optics, Hans-Kopfermann-Strasse 1, 85748 Garching, Germany

## Abstract

Visualizing the rearrangement of atoms in a wide range of molecular and condensed-matter systems requires resolving picometre displacements on a 10-fs timescale, which is achievable using pump–probe diffraction, given short enough pulses. Here we demonstrate the compression of single-electron pulses with a de Broglie wavelength of 0.08 ångström to a full-width at half-maximum duration of 28 fs or equivalently 12-fs root-mean square, substantially shorter than most phonon periods and molecular normal modes. Atomic resolution diffraction from a complex organic molecule is obtained with good signal-to-noise ratio within a data acquisition period of minutes. The electron-laser timing is found to be stable within 5 fs (s.d.) over several hours, allowing pump–probe diffraction at repetitive excitation. These measurements show the feasibility of laser-pump/electron-probe scans that can resolve the fastest atomic motions relevant in reversible condensed-matter transformations and organic chemistry.

## Abstract (web summary)

High spatial and temporal resolutions are required in order to follow chemical and condensed matter transformations in real time. Here, the authors compress single-electron pulses in time, with low jitter and high repetition rates, and demonstrate atomic resolution via diffraction from organic molecules.

## Graphical abstract

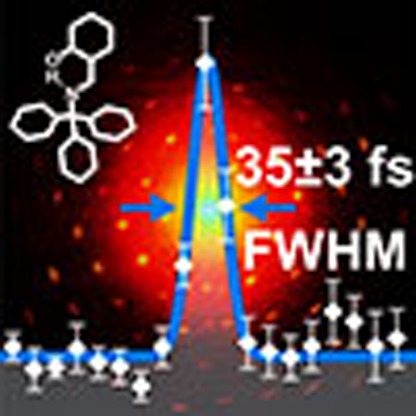

Chemical reactions and structural transitions in condensed matter are defined by the pathways of atomic motion in space and time^1^,^2^,^3^,^4^. The rapidity of these dynamics is dictated by the mass of the moving atoms and the restoring forces determined by the binding energy. The half periods of the normal modes in organic molecules and of the optical phonons in condensed matter typically last 10–20 fs or longer, defining the characteristic timescale of structural rearrangements. Wavepackets of light or matter of 10-fs-scale duration and picometre-scale wavelength are therefore required for capturing the entire dynamics in space and time for a slow-motion replay.

Time-resolved electron diffraction^4^,^5^,^6^,^7^,^8^,^9^,^10^,^11^,^12^,^13^ and microscopy^14^ constitute a cost-effective and complementary alternative to X-ray-based techniques relying on high-power lasers or particle accelerator facilities^15^,^16^ and offer more flexibility than laser-induced self-diffraction^17^,^18^ for the four-dimensional visualization of atomic-scale motions. Pioneering studies with ultrashort-pulsed electron beams provided new insight into chemical reactions^5^,^7^,^11^, melting processes^6^,^8^, surface dynamics^10^ or structural phase transformations^4^,^9^,^12^, complementing X-ray-based pump–probe investigations^19^,^20^ via the electrons' much higher scattering cross-section, subatomic de Broglie wavelength and direct energy tunability. However, the duration of the shortest single-electron or multielectron pulses reported so far^21^,^22^,^23^ is 67–170 fs (s.d.) or 160–400 fs (full-width at half-maximum, FWHM) and limits the temporal resolution of electron diffraction and microscopy to ≥100 fs, which is insufficient for resolving primary reaction paths entirely.

Here we demonstrate the compression of electron pulses to a root-mean-square duration of 12±2 fs or equivalently to a FWHM duration of 28±5 fs (errors as s.d.'s of the fits), a factor of six shorter than the briefest electron pulses demonstrated before^21^ and more than an order of magnitude shorter than used in previous time-resolved electron diffraction studies^4^,^5^,^6^,^7^,^8^,^9^,^10^,^11^,^12^,^13^. The laser-electron-timing stability is better than 5 fs (s.d.) over several hours, which allows accumulating pump–probe diffraction via reversible laser excitation. The de Broglie wavelength and beam coherence provide atomic resolution, demonstrated via Bragg diffraction from an organic molecular crystal. These combined achievements indicate that electron-based imaging technology now provides atomic resolution in space and time, provided reversible or replenishing reactions.

## Results

### Reversible and irreversible broadenings and their origins

In contrast to the photons in X-ray/optical pulses, electrons carry a charge. Coulomb forces therefore broaden multielectron pulses in time during propagation. This effect can be reduced by miniaturized diffraction geometries^2^, by reversing the linear chirp^24^ using microwave compression^21^,^22^,^23^,^25^,^26^, by photo-triggered streaking^27^ or by acceleration to MeV energies^28^,^29^,^30^. However, irreversible phase space broadenings are inevitable in multielectron pulses and prevent compression to quantum-limited combinations of duration, monochromaticity, beam size and coherence.

Single-electron pulses^31^ do not suffer from Coulomb repulsion and can, in principle, be shorter than 1 fs (refs 25, 26). However, vacuum is dispersive for electrons because of their rest mass, and temporal broadening of single-electron wavepackets arises from their finite energy bandwidth, as different-energy portions of the packet propagate with different speed. This lengthening mechanism is unavoidable. The initial pulse duration is dictated by the generation process, usually photoelectric emission; however, the stronger the temporal confinement of the emission, the broader is the energy bandwidth of the ejected electron wavepacket. This is a fundamental limit in addition to work function effects^32^. In feasible electrostatic acceleration fields, finite energy widths at optimum conditions limit the pulse duration to about a hundred femtoseconds (FWHM) after acceleration into the multi-keV range required for diffraction^33^. Although photoemission from nanometre-sized tips can result in extremely short emission times^34^,^35^ and near-tip acceleration enhancement^36^,^37^, the pulses nevertheless disperse and suffer from spatiotemporal distortions, leading to many tens to hundreds of femtoseconds (FWHM) during acceleration and collimation^36^,^37^. Pulses shorter than the duration of photoemission cannot be produced by any electrostatic acceleration concept.

### Single-electron compression by temporal energy modulation

Optical pulses are routinely compressed beyond their Fourier-limited duration by nonlinear spectral broadening followed by passage through a dispersive delay line. The first process generates new spectral components with a well-behaved, monotonic frequency sweep (chirp), which is removed in the second step by a tailored frequency-dependent delay. The concept relies on deterministic, highly reproducible mechanisms in both stages.

We adapt this general pulse compression concept to electron pulses carrying a few or a single electron by imposing a time-dependent energy modulation with a microwave acceleration field^25^,^26^. This is in contrast to other applications of microwave technology for electron pulse compression^21^,^22^,^23^, where Coulomb effects are addressed rather than the quantum effects of propagation. First, we create an electron wavepacket by femtosecond photoemission and let it disperse in time on passage through a static-field accelerator. Absence of space charge effects in the single-electron regime avoids the statistical variation of electron–electron interaction, and the process is therefore highly deterministic. The dispersion of vacuum leads to the buildup of a near-linear negative chirp with the low-energy components lagging behind the high-energy ones. Second, we apply a time-dependent longitudinal electric field to turn the negative chirp into a positive one while simultaneously increasing the energy bandwidth to provide room for shortening the duration below its initial value. Third, vacuum dispersion during propagation to a diffraction target eliminates the positive chirp and leads to temporal compression. The pulses become significantly shorter than the initial duration at photoemission, as defined by the laser pulses. Effectively, the energy spread increases in proportion to the compression in time, in accordance with Liouville's theorem.

**Figure 1:**
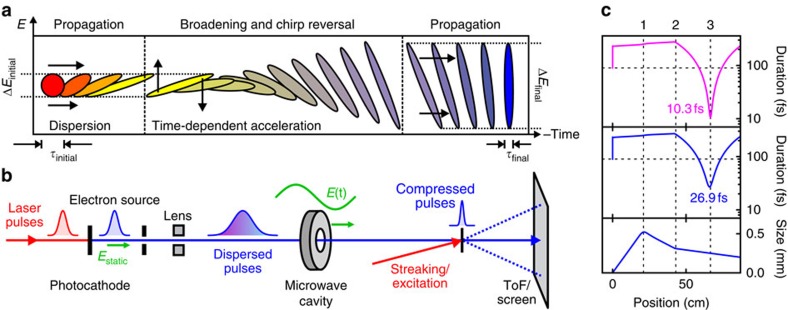
Temporal localization of single-electron pulses to arbitrarily short durations. (**a**) Phase space in the time–energy domain. Laser-generated subrelativistic single-electron pulses (red) at a duration of *τ*_initial_ disperse in time during acceleration and propagation (yellow). A time-dependent deceleration and acceleration (vertical arrows) causes a time-dependent energy modulation and gain in bandwidth. Simultaneously, the chirp is reversed (violet). Propagation in free space towards a diffraction target compresses the pulses to shorter than the initial duration (blue). The achievable final pulse duration is only limited by the tolerable increase in the energy spread from *Δτ*_initial_ to *ΔE*_final_. Since tens of eV are acceptable at tens-of-keV-ranged central energies, this concept allows making few-femtosecond and eventually attosecond electron pulses. (**b**) Experimental realization. Single-electron pulses are generated via laser pulses (red), photoelectric emission and electrostatic acceleration (*E*_static_, green). The dispersed pulses pass through a microwave cavity (grey) with oscillating longitudinal electric fields (*E*(t), green). Pulse characterization by laser-field streaking (red) with a time-of-flight spectrometer (ToF) and diffraction on a screen reveal the compressed pulse duration (*τ*_final_) and capability for atomic-scale imaging. (**c**) Particle-tracking simulations of the experimental geometry. Shown are the pulse duration of the on-axis electrons (pink) and the duration and diameter of the integrated beam profile (blue). Few-femtosecond pulse durations are achievable, although the initial electron generation takes 90 fs (dashed horizontal line). 1, magnetic lens; 2, microwave cavity; 3, temporal focus and diffraction sample.

Figure 1a depicts these three steps in the time–energy representation of the phase space for single-electron pulses. The ellipses schematically sketch the statistical distributions of propagation times and energies over a large number of single-electron pulses emitted and propagated under identical conditions^33^. An essential part of the concept is a much stronger temporal gradient of the electric fields than needed for just compensating the incoming single-electron dispersion. This deterministic broadening is analogous to the nonlinear spectral broadening of optical pulses and creates the precondition for temporal compression surpassing the initial pulse duration, as dictated by the conservation of the phase space volume (Liouville's theorem). In the absence of space charge, every ellipse in Fig. 1a must have the same area. If all interactions are sufficiently linear, that is, if the ellipses do not become curved or otherwise distorted, the compression in time is determined by the increase in energy bandwidth achievable or tolerable in the experiment. Practical single-electron sources have a bandwidth of ∼0.4 eV (ref. 38). A hundredfold broadening to tens of eV is easily tolerable at a central energy of many tens of keV. The resultant potential for a hundredfold temporal compression can in principle shorten electron pulses with an initial duration of tens of femtoseconds to durations of hundreds of attoseconds^25^,^26^,^39^.

### Experimental implementation

So far, this concept has been examined theoretically^25^,^26^ and with preliminary, indirect investigations^40^. Here we demonstrate the experimental realization (Fig. 1b). At the photocathode, we avoid (or strongly diminish) space charge by generating single-electron (or several-electron) pulses (blue) with attenuated ultraviolet laser pulses^41^. Electrostatic acceleration to 25 keV results in a de Broglie wavelength of 0.08 ångström. A solenoid is used to collimate the beam. The dispersed pulses (blue gradient) pass through an oscillating longitudinal electric field in a microwave cavity, which is described in detail in ref. 40. The zero crossing of the field is synchronized to the laser pulses using optically enhanced photodetection without a feedback loop^42^, and long-term drifts are corrected *in situ* using the electron energy spectrum; see Methods. After the cavity, the electron pulses impinge on a diffraction target (black) or streaking foil for pulse characterization^38^. A time-of-flight analyser measures the electron spectrum and a phosphor screen equipped with a camera records diffraction patterns.

We chose a practical distance of 24 cm between the cavity and the temporal focus, implying an energy broadening by several eV. The ultraviolet laser pulses used for electron generation have a duration of *τ*_initial_≈90 fs (FWHM) and the cavity increases the bandwidth from Δ*E*_initial_=0.4 eV to Δ*E*_final_=7.3 eV (FWHM). Assuming linearity of all phase space transformations (Fig. 1a), the considerations summarized above imply the potential of shortening the pulses to a duration of *τ*_final_=(Δ*E*_initial_/Δ*E*_final_)*τ*_initial_=4.9 fs (FWHM).

In realistic electron sources, the phase space after acceleration is slightly curved in the time–energy domain. In addition, different parts of the beam profile can suffer from slightly different delay. Figure 1c shows the results of simulations using General Particle Tracer (www.pulsar.nl/gpt) for the experimental geometry. Considering only electrons reaching the target on the optical axis^26^, the predicted FWHM pulse duration is 10.3 fs (pink curve). Integration across the full beam profile yields 26.9 fs (blue curves). Application of an ‘isochronic' magnetic lens^43^ avoids spatial curvature within the beam and would allow achieving compression across the entire beam profile without the need for a lossy pinhole, as proposed initially in ref. 26. For this case, our computations predict a full-beam pulse duration of 10.7 fs. This is only two times longer than the fundamental limitation imposed by the phase space volume, indicating that temporal nonlinearities and spatial distortions can be restricted to acceptable levels in properly designed geometries.

### Electron pulse characterization with an optical streak camera

We measure the duration, bandwidth and chirp of the compressed electron pulses using a recently reported laser streaking technique^38^. Briefly, the electrons pass through a metal foil (replacing the sample in diffraction experiments) while laser pulses are reflected, resulting in energy gain or loss from the laser field; see Methods. The laser-field-induced variation in electron energy is recorded as a function of delay between the electron and laser pulse, yielding what we refer to as a streaking spectrogram.

**Figure 2:**
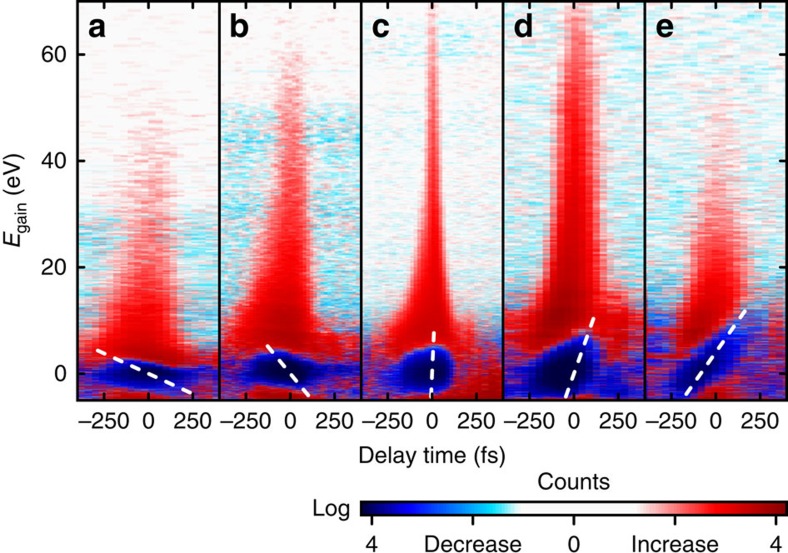
Characterization of compressed single-electron pulses by laser-field streaking. For an increasing microwave compression strength, five representative streaking spectrograms (**a**–**e**) show the expected reversal of chirp (white dashed lines) and pulse shortening to a few-femtosecond duration (**c**). From **a**–**e**, the compression strength *g*_E_ is 8.0, 14.2, 20.1, 25.3 and 31.9 eV ps^−1^, respectively; *E*_gain_, energy gain.

We obtained streaking spectrograms for a substantial range of amplitudes of the microwave peak field, oscillating at 6.2 GHz. This amplitude controls the rate of change of the energy transfer, *g*_E_ (in eV ps^−1^), from the field to the electron pulse. Five representative results are shown in Fig. 2, with the unstreaked electron spectra at large negative delays subtracted. Constant-energy-shift lineouts can be understood as a convolution of the electron pulse envelope with a ‘sampling' function given by the envelope of the laser field, truncated at a threshold corresponding to the selected energy shift^38^. The spectrograms also reveal the bandwidth and chirp of the electron pulses.

### Microwave-field-induced phase space transformations

**Figure 3:**
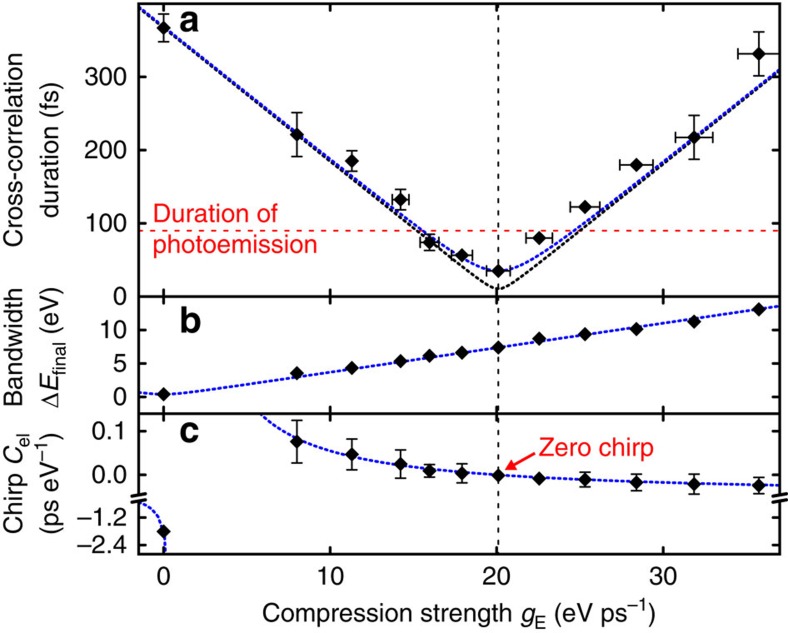
Measured and predicted pulse duration, bandwidth and chirp. (**a**) The width of the laser-electron cross-correlation (diamonds) shows a decrease and increase around an optimum compression strength at 20.1 eV ps^−1^ (dashed vertical line). There, the achieved pulse duration is shorter than the duration of the photoemission process used initially for electron generation (red dashed line). (**b**) The measured bandwidth of the compressed pulses (diamonds) shows the expected increase with the microwave compression strength. (**c**) The electron chirp (diamonds) is determined from the slope of the decrease feature at zero-energy gain in the streaking spectrograms (Fig. 2). Without any microwave field (0 eV ps^−1^), the chirp is negative (lower-energetic electrons arrive late) and resembles the yellow ellipse in Fig. 1a. For increasing compression by the microwave gradient, the chirp shows a reversal of sign and approaches zero for 20.1 eV ps^−1^. For stronger compression, the chirp becomes negative again, denoting an overcompressed electron pulse at the location of characterization. In all panels, error bars are s.e.'s and the dotted lines depict the results of a model prediction assuming linearity of the involved phase space conversions (see Methods). No fitting is applied.

Before evaluating the final electron pulse duration, we discuss the dynamics of the wavepacket transformation by the compression cavity and subsequent propagation. Figure 3 shows the laser-electron cross-correlation width, the electron bandwidth and the chirp (diamonds) for varying microwave compression strength *g*_E_ in comparison with a simple model calculation (blue dotted lines) assuming linearity of vacuum dispersion and microwave interaction in the time–energy domain; see Methods. The good agreement indicates that all involved phase space transformations perform in a linear way, as intended with the three key steps of our compression concept (deliberate dispersion, time-dependent energy modulation and self-compression towards the target).

### Shortest electron pulses

**Figure 4:**
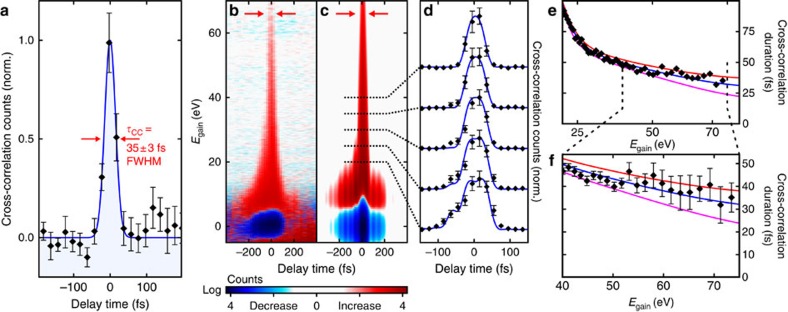
Shortest electron pulses. (**a**) The laser-electron cross-correlation, integrated for energy gains of 65–80 eV, has a width of 35±3 fs (FWHM). This measurement is limited by the duration of the streaking laser field; hence, the electron pulses are shorter. (**b**) Measured streaking spectrogram of the compressed pulses; the red arrows denote the lower-energy boundary of where the laser-electron cross-correlation in **a** is evaluated. *E*_gain_, energy gain. (**c**) Result of semiclassical simulations, fitted for electron pulse duration and third-order laser dispersion. (**d**) Sections at 20, 25, 30, 35 and 40 eV show a good agreement between measurement (diamonds) and simulation (blue). (**e**) Cross-correlation width (diamonds) for each *E*_gain_ in comparison with the simulation result assuming 18-fs (purple), 28-fs (blue) or 38-fs (red) electron pulses. (**f**) Streaking characterization is most reliable at highest energy gains^38^. The 28-fs pulses (colours as in **e**) have the best agreement to the measured data. All error bars are s.e.'s.

We now focus on the shortest electron pulses achieved experimentally, represented by the spectrogram recorded at *g*_E_=20.1 eV ps^−1^ (Fig. 2c). The cross-correlation width inferred from this spectrogram strongly depends on the energy gain, because the laser field responsible for streaking has a non-negligible, energy-dependent temporal extension of the field envelope on this scale^38^. Figure 4a depicts the measured laser-electron cross-correlation, integrated from 65 to 80 eV. A Gaussian fit shows a cross-correlation width of *τ*_C*C*_=35±3 fs FWHM; this cross-correlation marks an upper limit for the electron pulse duration.

To deconvolute the real electron pulse duration, we invoke semiclassical modelling of the streaking process, by combining Equation 4 of ref. 38 with a spatial integration across the electron and laser beam at the foil; see Methods. The streaking field is derived by Fourier transformation of the measured optical spectrum assuming zero lowest-order (linear) chirp, and the effective peak field is set equal to 2 × 10^9 ^V m^−1^, as used in the experiment. There are only two fitting parameters: the electron pulse duration and the third-order dispersion of the optical pulses. We find an optimum at 28 fs (FWHM) and −20,200 fs^3^, respectively. Figure 4b–d compares the simulated streaking spectrogram with the experimental data and reveals an excellent agreement. The measured energy-dependent cross-correlation widths are compared in Fig. 4e,f with simulation results for three different electron pulse durations, 18, 28 and 38 fs (FWHM). The 28-fs duration yields best agreement with the data and is tainted with an estimated uncertainty of ±5 fs. In terms of s.d., the pulses have a duration of 12±2 fs.

Notably, the pulses are significantly shorter than the duration of photoemission (90 fs FWHM, red dashed line in Fig. 3a) and shorter than any other event in the entire experiment, including fundamental Fourier-limited laser pulse durations. In the picosecond regime, some shortenings below the duration of photoemission have also been reported^44^,^45^, but with intentionally chirped photoemission pulses. In contrast, the presented single-electron time–energy reshaping technique can shorten the electron pulses beyond any initial duration limitations, analogous to optical pulse compression via nonlinear broadening and chirp compensation. The associated energetic broadening (here to 7.3 eV) is almost irrelevant at tens of keV central energy. The measured 28±5-fs electron pulse duration is slightly larger than the prediction of 26.9 fs (Fig. 1c), which is attributed to a residual jitter between the microwave field and the streaking pulses^40^ on the order of 10 fs, which is significantly better than elsewhere^23^,^46^ because of direct synchronization; see Methods.

### Effects of electron–electron interactions

**Figure 5:**
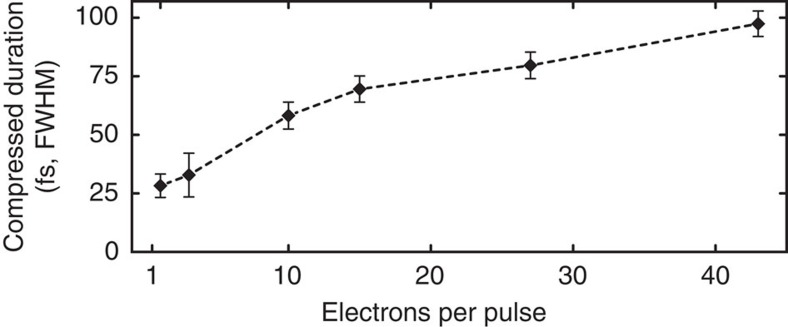
Effects of space charge. For more than one electron per pulse, the best achievable pulse duration increases as a consequence of statistical electron–electron Coulomb interaction. Error bars denote the s.e. from the de-convolved cross-correlation fits (compare Fig. 4).

The experiments reported above were carried out with genuine single-electron pulses, where space charge is nonexistent. To clarify to what extent our compression concept would also work for multielectron pulses, we repeated the above set of experiments with electron bunches containing an average number of 3, 10, 15, 27 and 43 electrons. In all cases, we found a clear minimum of the pulse duration when scanning the microwave amplitude, like in Fig. 3a. The required microwave energy transfer increases from *g*_E_=20.1 eV ps^−1^ for single-electron pulses to *g*_E_=25.3 eV ps^−1^ for 43 electrons per pulse. This increase reflects the space charge forces the cavity must work against in addition to compensating the wavepacket dispersion in vacuum. Figure 5 shows for each electron density the shortest achieved pulse duration. As expected, the pulse length increases for increasing the number of electrons per pulse, demonstrating the adverse effect of statistical electron–electron interactions (heating) on the achievable electron pulse compression.

Considering the strong repulsive forces acting between nearby electrons, it is quite remarkable that a bunch carrying 10 electrons and originating from a high-coherence source of few-micrometre size^41^ can still be compressed to a duration of 58 fs (FWHM), merely a factor of two longer than the minimum single-electron pulse duration. Even more electrons could be likewise compressed with a larger source size. This offers the possibility of a substantial reduction of experimental data acquisition time in time-resolved electron diffraction experiments by compromising temporal resolution by less than a factor of two, given that improved statistics may partially compensate for the increased probe pulse duration.

### Long-term stability

**Figure 6:**
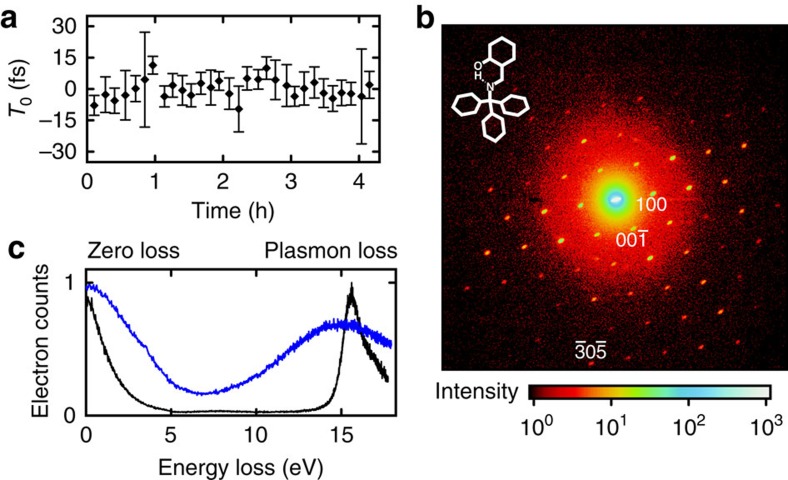
Diffraction and spectroscopy with compressed electron pulses. (**a**) Repeated laser-electron streaking measurements reveal a superior long-term stability of *T*_0_, the coincidence of laser pulses and electron pulses in time. The error bars are s.e.'s of the streaking spectrograms' time-zero fits. (**b**) Static diffraction pattern obtained with compressed electron pulses (28 fs FWHM) focused on a molecular crystal with unit cell dimensions of 1 nm. The measured intensities and sharpness of the spots demonstrate the ability to achieve atomic-scale resolution with compressed single-electron pulses. (**c**) Electron energy loss spectroscopy (EELS) of aluminium obtained with uncompressed (black) and compressed (blue) single-electron pulses. Although the compressed pulses (blue) are broadend according to Fig. 1, the plasmonic loss peak at ∼17 eV, representing the bulk metal's plasma frequency, is clearly distinguishable from the zero-loss peak in both cases (370- and 28-fs FWHM duration, respectively).

Ultrafast diffraction with single-electron pulses requires long-term timing stability between pump and probe pulses over many hours^47^. We achieve this by recording timing drifts of the microwave's phase via the corresponding change of the electron pulse's central energy^40^; see Methods. Figure 6a depicts an out-of-loop time-zero measurement of a series of streaking spectrograms. The s.d. of the timing drift is below 5 fs, which is a small fraction of the compressed electron pulse duration, demonstrating the feasibility of long-term diffraction measurements at sustained temporal resolution.

### Atomic resolution in diffraction

To verify the transverse coherence and capability of atomic resolution imaging with microwave-compressed single-electron pulses, we obtained static diffraction patterns from a molecular crystal of N-(triphenylmethyl)-salicylidenimine, which forms a bimolecular unit cell with a size of ∼1 × 1 × 1 nm^3^ (ref. 41). Freestanding 50-nm films were produced using ultramicrotomy and were placed at the temporal focus. Figure 6b shows the diffraction pattern with the spatial focus adjusted on the screen. Some asymmetry is due to imperfections in the angular alignment. The observed sharpness of the spots indicates that the superior transverse coherence of the original single-electron source^41^ is not impeded by the microwave compression and atomic resolution is achieved.

Femtosecond electron energy loss spectroscopy is another powerful method for time-resolved structural analysis^48^,^49^. Figure 6c shows the loss spectrum of the aluminium foil used for streaking, recorded with uncompressed (370 fs) and compressed (28±5 fs) electron pulses, respectively. The plasmon loss band at 17 eV is well discernible from the zero-loss peak in both cases, suggesting that time-resolved electron spectroscopy is another promising option for investigating matter transformations with the atomic-scale temporal resolution offered by the compression technique presented here.

### Signal-to-noise

Ultrafast electron diffraction implemented with single- or few-electron pulses relies on diffraction signal accumulation over millions to billions of pump–probe exposures and requires the highest repetition rate applicable to the diffraction target. This is ∼300–800 kHz for condensed-matter crystals, for example, graphite^47^ or vanadium dioxide^50^, and may be higher if less than one photon per unit cell can trigger the reaction in more complex materials^4^,^9^. The fastest atomic motions are encoded in changes of diffraction intensities and shot noise limits the experimental detection. In a previous report^47^, we had demonstrated the feasibility of pump–probe single-electron diffraction at the example of a graphite nanocrystal layer, a material where sub-30-fs dynamics is extremely important (strongly coupled optical phonons). We had used uncompressed single-electron pulses with the same tens-of-nm coherence, picometre de Broglie wavelength, micrometre-sized beam and atomic resolution capability as applied here for compression. Few-percent relative intensity noise of Bragg spots had been achieved, which is comparable to state-of-the-art with brighter/longer electron pulses at lower repetition rate^51^. For the N-(triphenylmethyl)-salicylidenimine crystal (Fig. 6b), the 10 most intense Bragg spots have count rates of 170–550 electrons/s with compressed single-electron pulses at 2.5-MHz repetition rate. Focusing on the sample^52^ produces larger Bragg spots with 3,000–7,000 electrons/s; see Methods. Assuming a realistic 300-kHz pump–probe repetition rate and 15-min integration time, this corresponds to 10^4^–10^6^ accumulated electrons per Bragg spot and pump–probe image. Shot noise is below 1% for such signals; this is sufficient for identifying sub-ångström atomic motion^4^,^53^. Whether laser excitation is reversible enough in that particular molecule remains to be determined (see Methods); however, these estimations and our previous experiential realization^47^ show that pump–probe single-electron diffraction is feasible at atomic resolution. A precondition is reversibility of the reaction or a sample that can be replenished.

## Discussion

The rapidity of the fastest structural rearrangements in molecular systems and condensed matter is dictated by the half period of normal modes and optical phonons, respectively. The half periods of stretch oscillations of C–C, C–O, C–N and similar bonds last 10–20 fs, whereas the normal modes promoting chemical reactions usually involve more than two atoms and hence have longer periods. The fastest known reactions occur during 30–100 fs (ref. 3). In condensed matter, the half periods of optical phonons range down to 20 fs, defining the fastest paths towards structural phase transformations. For example, tens-of-fs dynamics is probably decisive for charge–density wave transitions^9^, vanadium dioxide^4^,^12^, graphite^13^, carbon nanotubes^54^ and some molecular switches^55^. With reasonable statistics, the demonstrated FWHM pulse duration of 28±5 fs offers a temporal resolution below 20 fs, sufficiently short for capturing the entire range of possible atomic motions in condensed matter and molecules, in addition to the slower 100-fs dynamics which is also important^56^.

Pump–probe experiments with single/few-electron pulses require diffracted signal accumulation over many consecutive pump–probe exposures under identical experimental conditions. In condensed-matter reactions, this condition is typically fulfilled^4^,^47^, while many molecules are susceptible to collateral reactions even if the major structural changes under scrutiny are reversible. For studying irreversible dynamics^2^ with few-electron pulses, a rapid replacement of molecular samples during data acquisition and their rapid alignment may therefore be necessary, which can be achieved with molecular beams^57^ and short-pulse lasers^58^, respectively. The single-electron pulses will be directly applicable to condensed matter at reversible operation conditions, which is the regime required for numerous applications, for example, metal-to-insulator switching^4^,^12^, solar energy conversion with activated surfaces^59^ or photocatalysis^60^. The demonstrated electron pulses combine sub-phonon duration, subatomic wavelength, nanoscale coherence, superior pump-pulse synchronization and a reasonable flux. This provides a solid basis for imaging almost any substance that can be reversibly excited or replenished between pump-pulse exposures.

The demonstrated ability to make single-electron wavepackets shorter than any other events in the experiment opens up another (long-term) perspective. In principle, electron pulses can be made shorter than the wave cycle of visible/infrared light for directly imaging light-driven electronic motion. This may provide unprecedented insight into the motion of electron densities on the scale of the lattice period in response to optical fields^39^,^61^ and pave the way to explore the ultimate limits of electronics^62^,^63^. The microwave compression technique demonstrated here, when implemented with subfemtosecond synchronization^64^ and isochronic imaging^43^, seems an approach worth considering towards that regime. At the present stage, the electron pulses are short enough to resolve *any*, in particular the fastest, atomic motion that is relevant in chemistry and condensed-matter transitions, given that a significant amount of the reaction can be effectively and reproducibly excited with pump pulses.

## Methods

### Laser-driven electron diffractometer and molecular-crystal sample

The laser is a Ti:Sapphire long-cavity oscillator producing 2.5 W of 60-fs, 800-nm pulses at 5.1-MHz repetition rate. A pulse picker reduces this to 0.1–2.5 MHz depending on the application. Electrons are detected with a quantum efficiency of nearly 100% using a phosphor screen and camera^47^. Single-crystalline freestanding layers of N-(triphenylmethyl)-salicylidenimine are produced by ultramicrotomy and are mounted on densely spaced transmission electron microscopy grids (2000 lines per inch)^41^. Diffraction is either produced by focusing the electron beam on the screen (highest coherence) or on the sample (highest yield)^57^. Fluorescence spectroscopy from the nm-thick layer^65^ revealed the expected ultrafast proton transfer via a huge Stokes shift; however, efficient pump–probe diffraction would require sub-20-fs ultraviolet excitation pulses at 340 nm, which are currently not available.

### Temporal pulse characterization

The streaking foil is a freestanding 50-nm-thick aluminium film, tilted at an angle of ∼17° for phase matching^38^. The electron beam diameter is 103 μm and the size of the laser focus is 56 × 86 μm FWHM. The spectrogram of the shortest pulse has a cutoff, that is, transition from measurable counts into noise, at 80±4 eV, in good agreement with the theoretical value of 77.4 eV obtained for a peak electric field of the streaking laser in the direction of the electron beam at the foil's surface of 2 × 10^9 ^V/m (ref. 38).

### Laser-electron synchronization

The critical step in laser-microwave synchronization is the precise detection of the laser timing. We therefore enhance the optical pulse train at a 5.1-MHz repetition rate with a Fabry–Pérot filter^42^, adjusted to the microwave cavity's centre frequency of 6.237 GHz. After photodetection, a single microwave mode of the enhanced frequency comb is filtered and amplified to ∼2 W. A variable attenuator and mechanical phase shifter are used to optimize the microwave gradient for compression. There is no feedback loop. We characterized this passive synchronization scheme with a fibre-loop optical mixer^64^ and found an integrated jitter of less than 5 fs (s.d.) in the range of 2 mHz to the Nyquist frequency, 2.5 MHz. The proximity between measured (28 fs) and simulated (26.9 fs) pulse durations supports this result. The achieved quality of synchronization exceeds reported values from phase-locked-loop electronics^23^,^46^ by a factor of ∼10.

### Drift correction

Single-electron diffraction^47^ necessitates superior long-term stability of the laser-microwave timing. Since the cavity's intrinsic phase can drift by thermal effects, an *in situ* approach is essential^46^. We draw on the proportional relation between the microwave phase and the central energy of the compressed pulses^40^: phase delays in the cavity cause a shift of the electron energy. Time-of-flight spectra are continuously acquired at a rate of ∼0.1 Hz and used to post correct the streaking spectra via their time stamp^66^, using the previously calibrated microwave energy transfer value *g*_E_ (ref. 40). The time-zero measurements in Fig. 6a provide an out-of-loop confirmation of that approach's final performance for laser-electron synchronization at the sample location.

### Phase space transformation model

Liouville's theorem on the conservation of phase space applies to the position-momentum domain; however, at limited bandwidths around 25 keV, changes in momentum are proportional to changes in energy and changes in position are proportional to changes in time. At *E*_0_=25 keV, the electrons have a velocity of *v*_0_≈0.3*c* and the dispersion of vacuum is 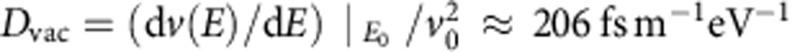. Without the microwave, the dispersed electron pulses have a bandwidth of *ΔE*_initial_=0.4 eV (FWHM), a duration of *τ*_disp_≈370 fs (FWHM) and a chirp of *C*_disp_=−0.9 ps eV^−1^ before the cavity (yellow pulse in Fig. 1a). The streaking foil, where the temporal focus should be, is located at a distance *f*=24±0.5 cm behind the microwave cavity. The predicted temporal focus^40^ is at 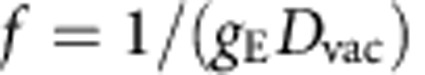. This yields *g*_E_=20.2±0.4 eV ps^−1^, which fits well to the experimental value of 20.1 eV ps^−1^ (Fig. 2c). Neglecting *ΔE*_initial_, the bandwidth *ΔE*_final_ of the electron pulse is given by *ΔE*_final_=*|τ*_disp_*g*_E_| (blue dotted line in Fig. 3b).

The chirp of the electron pulses, *C*_el_, at the streaking target depends on three components: the initial chirp *C*_disp_ before the cavity, the effect of energy modulation in the microwave cavity (*g*_E_) and the subsequent temporal compression through free propagation (*D*_vac_). The cavity modulates the chirp in the energy domain, while vacuum dispersion modulates in the time domain. We obtain 
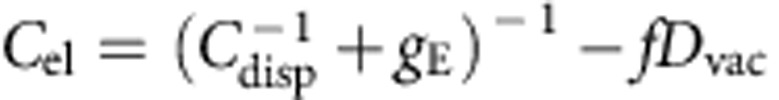
. The result (blue dotted line in Fig. 3c) shows the expected reversal of sign and the chirp becomes zero for *g*_E_=20.1 eV ps^−1^, denoting the compressed pulse. The duration at the streaking target is 
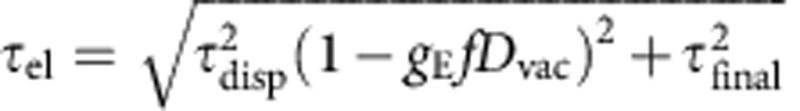
. The black dotted line in Fig. 3a shows the result assuming *τ*_final_=10.3 fs (ideal compression; see Fig. 1c). However, the experimental values depicted in the same panel are cross-correlation widths; the blue dotted line shows *τ*_el_ assuming a minimum measurable cross-correlation width of *τ*_final_=*τ*_CC_=35 fs (Fig. 4a), limited by spatial beam distortions, laser-microwave jitter and the streaking pulse's finite duration.

## Additional information

**How to cite this article:** Gilserin, A. *et al*. Sub-phonon-period compression of electron pulses for atomic diffraction. *Nat. Commun*. 6:8723 doi: 10.1038/ncomms9723 (2015).
